# Pemafibrate (K-877), a novel selective peroxisome proliferator-activated receptor alpha modulator for management of atherogenic dyslipidaemia

**DOI:** 10.1186/s12933-017-0602-y

**Published:** 2017-10-04

**Authors:** Jean-Charles Fruchart

**Affiliations:** R3i Foundation, St. Alban-Anlage 46, Basel, Switzerland

**Keywords:** Peroxisome proliferator-activated receptor alpha, SPPARM, K-877, Pemafibrate, Fibrates, Residual cardiovascular risk, Triglycerides, Atherogenic dyslipidaemia

## Abstract

Despite best evidence-based treatment including statins, residual cardiovascular risk poses a major challenge for clinicians in the twenty first century. Atherogenic dyslipidaemia, in particular elevated triglycerides, a marker for increased triglyceride-rich lipoproteins and their remnants, is an important contributor to lipid-related residual risk, especially in insulin resistant conditions such as type 2 diabetes mellitus. Current therapeutic options include peroxisome proliferator-activated receptor alpha (PPARα) agonists, (fibrates), but these have low potency and limited selectivity for PPARα. Modulating the unique receptor–cofactor binding profile to identify the most potent molecules that induce PPARα-mediated beneficial effects, while at the same time avoiding unwanted side effects, offers a new therapeutic approach and provides the rationale for development of pemafibrate (K-877, Parmodia™), a novel selective PPARα modulator (SPPARMα). In clinical trials, pemafibrate either as monotherapy or as add-on to statin therapy was effective in managing atherogenic dyslipidaemia, with marked reduction of triglycerides, remnant cholesterol and apolipoprotein CIII. Pemafibrate also increased serum fibroblast growth factor 21, implicated in metabolic homeostasis. There were no clinically meaningful adverse effects on hepatic or renal function, including no relevant serum creatinine elevation. A major outcomes study, PROMINENT, will provide definitive evaluation of the role of pemafibrate for management of residual cardiovascular risk in type 2 diabetes patients with atherogenic dyslipidaemia despite statin therapy.

## Background

Preventing cardiovascular disease (CVD) is a key challenge facing clinicians world-wide this century. Despite improvement in CVD mortality in developed regions, low to middle-income countries have a growing burden of CVD mortality and disability, driven by increasing rates of obesity, diabetes and dyslipidaemia [1–3]. Indeed, with the exception of the USA, the top 10 countries for diabetes prevalence in 2040 will be emerging economies, led by China and India [4]. Consequently, much of the future societal burden of CVD, estimated to exceed one trillion dollars by 2030 [5], will be in regions which can ill afford such costs.

Low-density lipoprotein cholesterol (LDL-C) is undoubtedly the primary priority for lipid management to prevent CVD [6, 7]. Statins, the cornerstone of LDL-C lowering therapy, have proven efficacy in reducing the risk of a CVD event by 20–30%, but still leave a high residual cardiovascular risk [8]. In part, this may be reduced by further lowering of LDL-C with non-statin therapy, as shown by the Examining Outcomes in Subjects With Acute Coronary Syndrome: Vytorin Ezetimibe/Simvastatin) vs Simvastatin (IMPROVE-IT) trial with ezetimibe [9], and the Further Cardiovascular Outcomes Research With PCSK9 Inhibition in Subjects With Elevated Risk (FOURIER) trial with evolocumab [10]; however, there is also the need to consider other lipid abnormalities. A growing body of evidence highlights the relevance of atherogenic dyslipidaemia, characterised by increased triglyceride (TG)-rich lipoproteins and their remnants (for which elevated TGs are a metric), often with subnormal plasma concentrations of high-density lipoprotein cholesterol (HDL-C), and an increase in small dense LDL particle numbers, typically seen in insulin resistant conditions such as type 2 diabetes mellitus, as a contributor to lipid-related residual cardiovascular risk, as well as the risk of silent coronary artery disease [11–13]. Overproduction of very low-density lipoproteins (VLDL), with increased secretion of TGs and apolipoprotein (apo) B100, appears to be a key driver of this dyslipidaemia.

Inhibition of cholesteryl ester transfer protein (CETP), which mediates the heteroexchange of triglycerides from VLDL or LDL and cholesteryl esters from HDL, was considered a possible therapeutic approach. However, the first three CETP inhibitors had either safety issues (torcetrapib) or no effect on clinical cardiovascular outcomes in high-risk patients (dalcetrapib, evacetrapib) [14–16]. The last of these agents, anacetrapib, was very recently shown to have a modest albeit statistically significant benefit over 4 years in the Randomized EValuation of the Effects of Anacetrapib Through Lipid-modification (REVEAL) trial, although the role of HDL-raising in this outcome remains a matter for scientific conjecture [17]. There has also been a renewed focus on the management of elevated TGs and remnant cholesterol (the cholesterol contained in TG-rich lipoproteins). Support for the atherogenicity of remnant cholesterol is based on the totality of evidence from observational studies showing an association between elevated remnant cholesterol and ischaemic heart disease, as well as genetic insights which show that remnant cholesterol is causal for ischaemic heart disease independent of HDL-C [18, 19]. Most recently, remnant cholesterol has been implicated as a contributor to the increased CVD risk associated with obesity [20]. These findings have prompted a rethink of the role of lipid targets beyond LDL-C, with for example, the American Diabetes Association recommending TG-lowering as an important secondary target in patients with diabetes [21]. It should be noted, however, that for isolated severe hypertriglyceridaemia, intervention is indicated due to pancreatitis risk not for prevention of CVD [22].

With growing recognition of the importance of targeting atherogenic dyslipidaemia comes a re-evaluation of available therapeutic options. Fibrates, peroxisome proliferator-activated receptor (PPAR) α agonists, are the most logical option as an add-on to statin therapy for management of elevated TGs and atherogenic dyslipidaemia, typically associated with insulin resistant conditions [7, 23]. While evidence in both primary and secondary prevention settings is supportive of their use [24, 25], the lack of a definitive mortality benefit has led many clinicians to view these agents as ‘second choice’. Despite this, there is support from subgroup analyses of the major fibrate trials that targeting these treatments to patients with atherogenic dyslipidaemia significantly reduces CVD risk (with or without statin treatment) [26]; the ACCORDION study, involving passive extended follow-up of the Action to Control Cardiovascular Risk in Diabetes (ACCORD) study, showed that this benefit persisted over the long-term [27]. Additionally, the Bezafibrate Infarction Prevention (BIP) study showed long-term benefit in reducing mortality, more so in individuals with baseline hypertriglyceridaemia [28].

With escalating rates of diabetes, obesity and CVD, especially in developing regions, management of atherogenic dyslipidaemia, as well as metabolic abnormalities and chronic inflammation typically associated with these conditions, is now a priority for reducing residual cardiovascular risk. This scenario highlights the need for new therapeutic options. Selective PPARα modulators–SPPARMα agents—could well provide one approach to resolving this challenge, and offer a more accessible therapeutic approach to managing this dyslipidaemia [29].

### Search strategy

This review describes the profile of a novel SPPARMα, determined using a search methodology as follows. The literature was searched using Medline, Current Contents, PubMed, and relevant references with the terms ‘residual cardiovascular risk’, ‘peroxisome proliferator-activated receptor’, ‘pemafibrate’, ‘K-877’, ‘atherogenic dyslipidaemia’, ‘triglycerides’, ‘type 2 diabetes’, ‘clinical trials.’ Main articles published in English between 2000 and 2017 were reviewed and included. The author also requested information regarding clinical trials and preclinical studies with pemafibrate from Kowa Company, Ltd., Japan.

## New thinking: the rationale for SPPARMs

PPARs are nuclear hormone receptors which bind to DNA as a heterodimer with the Retinoid X Receptor (RXR), and together they recognise specific DNA sequences in and around target genes referred to as PPAR response elements (PPREs). There are three different types of PPARs, ɑ, β and γ [30]. PPARα is abundantly expressed in highly active metabolic tissues such as the liver, kidney, heart, muscle, brown adipose tissue, as well as the vascular wall (smooth muscle cells, endothelial cells and macrophages). In contrast, PPARγ is predominantly expressed in white and brown adipocytes, macrophages and the large intestine, and PPARβ in virtually all tissues and cell types [23, 30, 31].

When activated by the binding of either an endogenous ligand or synthetic PPAR agonist (such as a fibrate for PPARα), heterodimerisation with a ligand-activated RXR results in a conformational change, leading to the transrepression or transactivation of target genes. During transrepression, the activated PPAR binds to cytokine-activated transcription factors, such as nuclear factor kappa B or activator protein-1, and blocks the interaction between the activated transcription factors and the promoter region of the target gene, thereby preventing transcription and, in this example, reducing inflammation. In contrast, during transactivation, the activated PPAR binds to PPRE upstream of the target gene, and with the involvement of cofactors, renders the PPAR complex ‘transcriptionally active’ [23, 29, 32–35]. A large number of genes carry response elements for PPARs.

The focus of interest in this review, PPARɑ, plays a key role in metabolic homeostasis, regulating lipid metabolism, specifically HDL synthesis and metabolism, and VLDL turnover, by controlling the expression of key targets including apo A-I, A-II, A-V and C-III, lipoprotein lipase (LPL), scavenger receptor B1 (SR-B1), the ATP-binding cassette transporters ABCA1 and ABCG1, and acyl CoA synthetase [23, 35, 36]. There is also evidence to suggest that pharmacological PPARα activation may be involved in regulation of glucose homeostasis (although the underlying mechanisms in humans are unclear), as well as reduction in inflammation and thrombogenesis, and improvement of vascular function [23, 34–36]. Thus, activation of PPARɑ results in attenuation of abnormal lipid and/or glucose metabolism, and is also protective against atherothrombosis by down-regulating inflammatory genes of monocytes and macrophages [23, 32, 33]. PPARα is therefore at the cross-roads of obesity, diabetes and CVD, and thus a logical target for therapeutic intervention. In contrast, PPARγ targets include genes involved in obesity and insulin resistance, and thus regulates adipogenesis and glucose homeostasis.

**Fig. 1:**
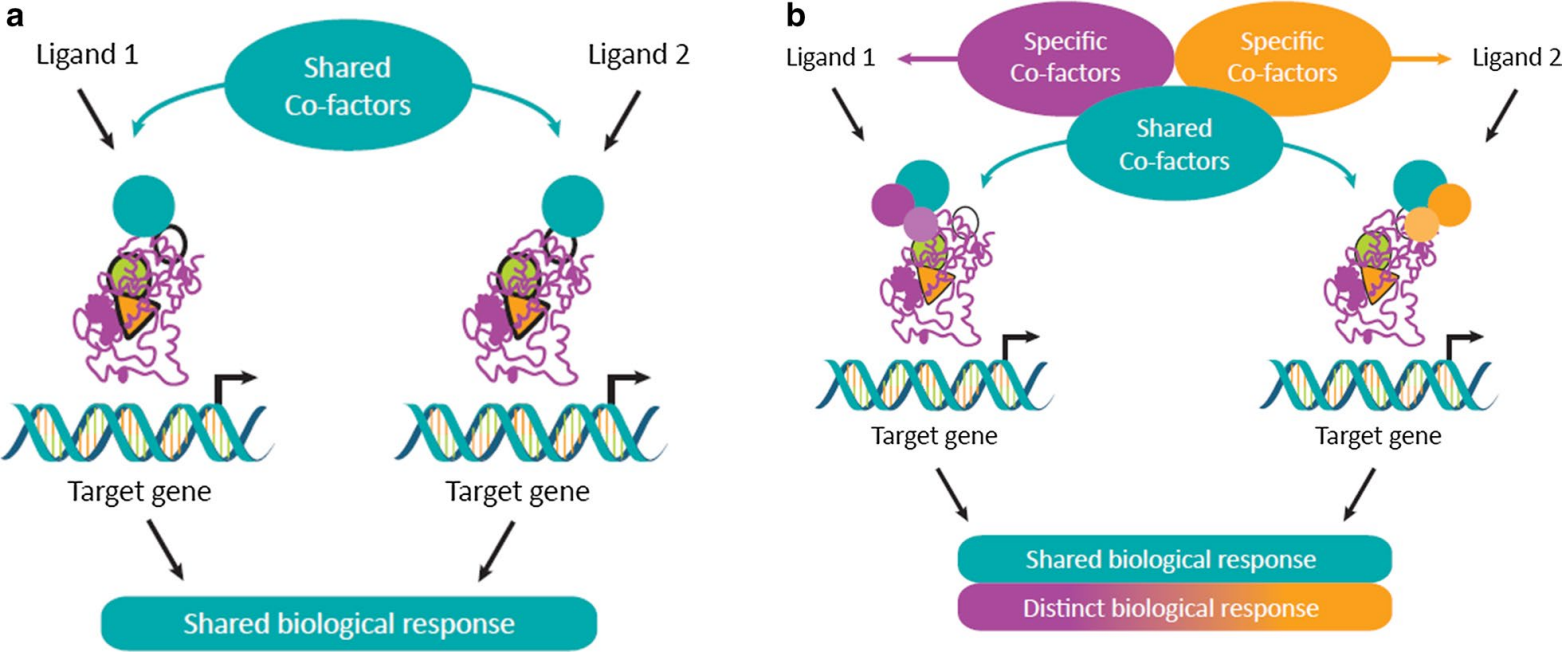
Schematic representation showing how selective nuclear receptor modulation underpins the SPPARM concept. The binding of different ligands to nuclear receptors induces different conformational changes which influence cofactor affinity. Different ligands may share cofactors, resulting in shared biological responses (**a**) but may also have distinct differences in the cofactor-receptor binding profile (**b**). Thus, the unique receptor-cofactor binding profile of the ligand is the key determinant of the specificity and potency of receptor binding and in turn modulates gene- and tissue selective effects

PPARs possess a large lipid-binding pocket capable of encompassing a range of endogenous ligands. On binding, each ligand triggers a unique conformational change,leading to differential pattern of coactivator recruitment, which in turn results in specific tissue- and gene-selective effects. It is important to note that while PPAR ligands may share cofactors leading to a shared biological response, there are also differences in cofactor selectivity, resulting in differing responses (Fig. 1). Thus, modulating the receptor–cofactor binding profile of the PPAR ligand offers the opportunity to improve desirable biological effects (via transactivation of desirable target genes), and limit known adverse effects (via transrepression of undesirable genes) of the PPAR ligand. This concept underlies the rationale for the development of SPPARMs which differentially induce a unique receptor-cofactor binding profile conferring improved efficacy and avoidance of unwanted side effects [37].

The SPPARM concept has already been applied to PPARγ. The first such SPPARMγs, INT131 and MK0533, have been shown in preclinical studies to exhibit at least comparable antidiabetic effects to pioglitazone but with an improved adverse event profile [23, 38, 39]. The SPPARM concept has also been recognized for PPARα. Indeed, there was early evidence for this as regulation of human apoA-I by gemfibrozil and fenofibrate was mediated by selective modulation of PPARα [40]. From the clinical perspective, the development of a novel SPPARMα with increased selectivity, high potency, as well as an improved safety profile compared with current PPARα agonists offers advantages in patients with atherogenic dyslipidaemia.

## A novel SPPARMα: pemafibrate (K-877)

**Fig. 2:**
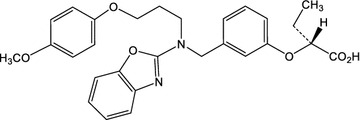
Structure of pemafibrate (K-877)

Pemafibrate (K-877, Parmodia™^)^) was identified as an agonist with very high potency and selectivity against human PPARα as a result of screening based on the SPPARMα concept. Pemafibrate differed from other PPARα agonists currently available, which were developed without definitive knowledge of their specific mechanism of action. Structurally, pemafibrate (K-877) has an acidic region as in other PPARα agonists, but with the addition of unique benzoxazole and phenoxyalkyl side-chains, resulting in greatly enhanced PPARα activity and selectivity [41] (Fig. 2). In cell-based transactivation assays, pemafibrate was shown to be > 2500-fold more potent than fenofibric acid, the active metabolite of fenofibrate, for human PPARα with > 5000-fold greater activity for PPARα than either PPARγ or δ [42].

Some characteristics of the gene transactivation profile for pemafibrate suggest that it is not just a PPARɑ agonist but a SPPARMɑ agent. Comprehensive transcriptome analysis showed that 11 of the top 20 genes up-regulated by pemafibrate were involved in carbohydrate and lipid metabolism, as for fenofibric acid. Key target genes such as *VLDLR* and *ABCA1* were induced to a greater extent by pemafibrate than fenofibric acid, but pemafibrate had no effect on peroxisome biogenesis genes in human hepatocytes [43]. Pemafibrate also rescued interferon γ-induced suppression of nuclear receptor co-repressor 1 and 2 (NCoR1 and NCoR2), co-repressors of pro-inflammatory cytokines, in macrophages, and suppressed pro-inflammatory mediators, including vascular cell adhesion molecule 1 (VCAM-1) and monocyte chemoattractant protein-1 (MCP-1) in endothelial cells. Anti-inflammatory effects were observed in human umbilical vein endothelial cells with pemafibrate 0.1 μM whereas fenofibrate at concentrations up to 10 μM had no effect [44, 45].

Other important differences were evident. Pemafibrate (but not fenofibric acid) upregulated the genes encoding mannose-binding lectin 2 (*MBL2),* involved in regulation of the innate immune system, inflammation and, possibly, vascular complications in diabetes [46, 47], as well as glutamyl aminopeptidase (*ENPEP*), involved in regulation of blood pressure [48], in transcriptome analysis [43]. In addition, pemafibrate induced expression of fibroblast growth factor 21 (*FGF21)* to a greater extent than fenofibric acid [43]. FGF21 has been implicated in the regulation of glucose, lipid and energy homeostasis in man, although findings are conflicting [49, 50]. There may also be a plausible biological link with non-alcoholic fatty liver disease (NAFLD), given that FGF21 decreases TGs, improves insulin sensitivity and counters obesity by suppressing weight gain, the major risk factor for NAFLD [49, 51]. These findings implicate a cooperative mechanism, possibly involving the combination of PPARα, cyclic AMP responsive element-binding protein (CREBH), and 3-hydroxy-3-methylglutaryl-CoA synthase 2 (HMGCS2), in regulation of FGF21 [52, 53]. Taken together, in vitro evidence for pemafibrate showed enhanced potency and selectivity for PPARα, suggesting added potential for management of dyslipidaemia.

## Preclinical data

Preclinical studies have established the pharmacological profile of pemafibrate, showing enhanced TG lowering and elevation in HDL-C levels compared with fenofibrate. In a rat model of hypertriglyceridaemia, the effect of pemafibrate 3 mg/kg on TG lowering was significantly greater (by about 2-fold) compared with fenofibrate (300 mg/kg), and was also accompanied by a greater increase in plasma levels of FGF21 (*Data presented at the 80th European Atherosclerosis Society Congress. May 25*–*28, 2012. Milan, Italy, personal communication from Kowa Company, Ltd*). In vitro studies using liver samples from Zucker Fatty rats showed that pemafibrate reduced de novo synthesis of TGs and cholesterol, and increased beta-oxidation. Sprague–Dawley rats dosed with pemafibrate showed inhibition of VLDL secretion and accelerated TG clearance via LPL activation [42]. Additionally, in C57BL/6J mice fed a Western diet, pemafibrate attenuated fasting and postprandial hypertriglyceridaemia, as well as accumulation of remnant lipoproteins, by enhancing LPL activity and reducing weight gain [54].

**Fig. 3:**
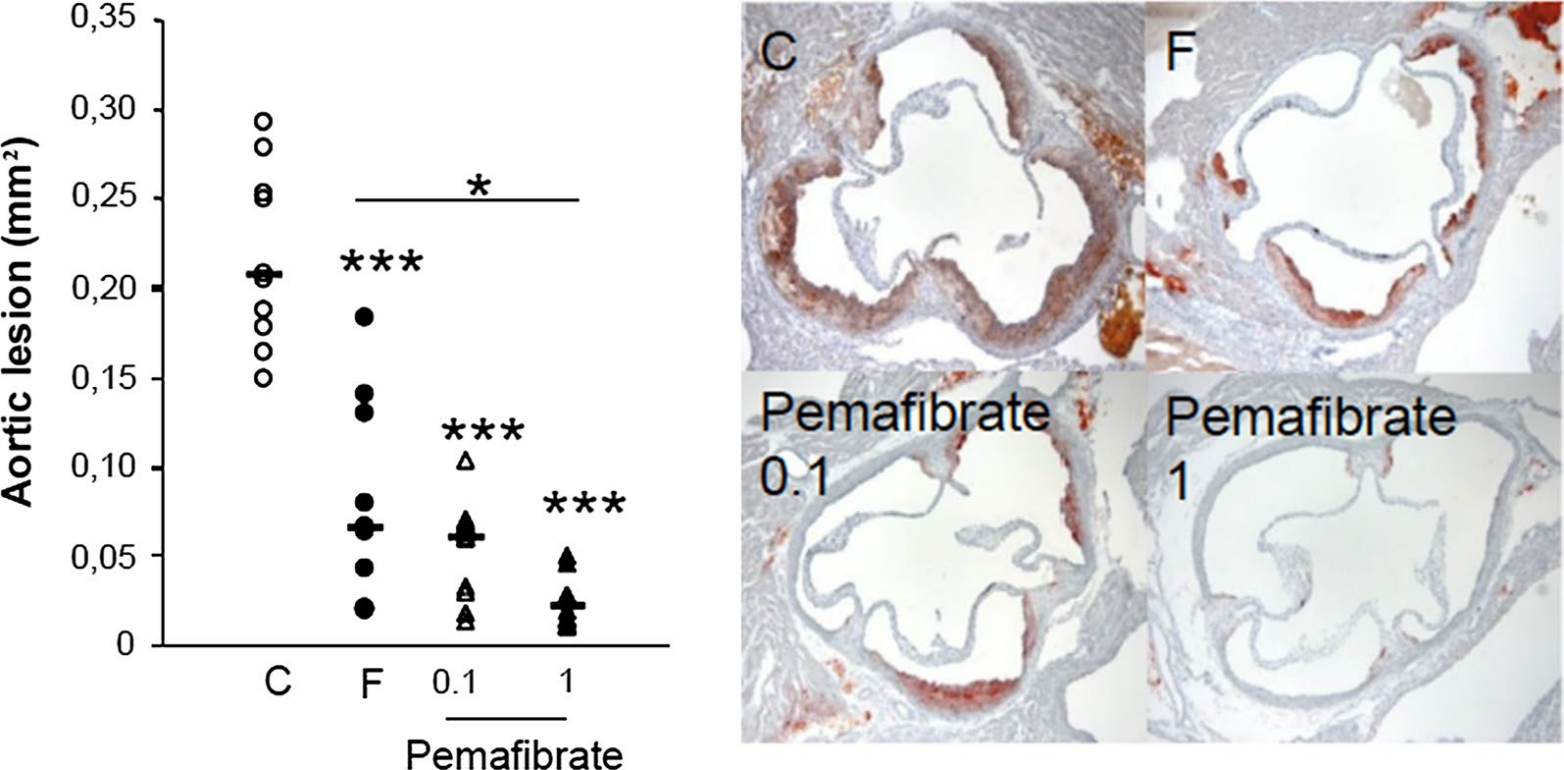
Anti-atherogenic effects of pemafibrate (K-877) in apolipoprotein E transgenic mice. ApoE2KI mice were fed a Western diet and treated with pemafibrate (0.1 or 1 mg/kg), fenofibrate (250 mg/kg) or control (carboxy methyl cellulose) daily and were sacrificed after 10 weeks. The left panel shows significant reduction in the atherosclerotic lesion area in mice dosed with pemafibrate 0.1 mg/kg compared with control. This effect was enhanced in the pemafibrate 1 mg/kg group. Each symbol represents the average area staining in the aortic sinus of individual animals and the bar represents the median of the values (n = 10 per group). *p < 0.05, ***p < 0.001 versus control. Representative photomicrographs showing Oil-red-O stained fatty-streaks in the atherosclerotic lesions is shown on the right panel. Reproduced with permission from Hennuyer et al. [55]

Administration of pemafibrate to transgenic apoE2 mice led to more pronounced HDL-C elevation at a 100-fold lower dose than fenofibrate (1 mg/kg versus 100 mg/kg) [*Data presented at the 80th European Atherosclerosis Society Congress. May 25*–*28, 2012. Milan, Italy, personal communication from Kowa Company, Ltd*]. Pemafibrate promoted macrophage cholesterol efflux to HDL in vitro, resulting in inhibition of lipid deposition in the aorta and reduction in the aortic atherosclerotic lesion burden in Western diet-fed apoE2KI mice compared with control; in contrast, the effect with fenofibrate was not statistically significant (Fig. 3) [55]. There was also evidence that the potent anti-inflammatory effects of pemafibrate observed in vitro translated to attenuation of atherosclerotic lesion development after mechanical injury in another animal model [44, 45, 55].

In summary, preclinical data show that pemafibrate modulated gene expression mediated by PPARα, which in turn led to improved beneficial effects on atherogenic dyslipidaemia, inflammation and atherosclerosis, when compared with current PPARα agonists. There was also evidence to suggest the possibility of novel targets, notably FGF21.

## Clinical trials

Pemafibrate is being investigated in a comprehensive clinical trial programme, initially in Japan and latterly in Europe/USA, as both monotherapy and add-on to statin therapy. To date, over 2300 patients, the majority with dyslipidaemia and over one-quarter with concomitant type 2 diabetes mellitus, have been studied [*Data on file, Kowa Company, Ltd.*]. Key published trials are summarised in Table 2. Pemafibrate has been approved for the treatment of dyslipidaemia in Japan (July, 2017), and is currently in Phase III development in Europe/USA.

## Efficacy

### Lipid effects

**Fig. 4:**
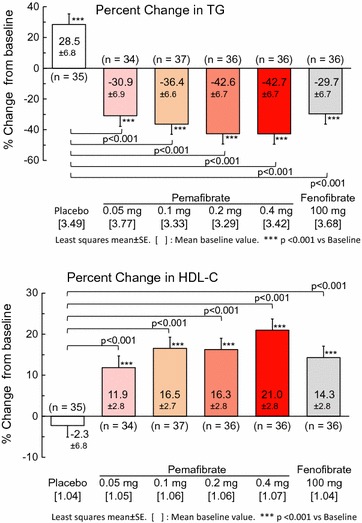
Least squares mean percent change in triglycerides (TG; top panel) and high-density lipoprotein cholesterol (HDL-C) (bottom panel) after 12 weeks treatment with pemafibrate (0.05, 0.1, 0.2 or 0.4 mg/day), fenofibrate (100 mg/day) or placebo in patients with elevated TG (≥ 200 mg/dL or 2.3 mmol/L) and low HDL-C (Adapted from Ishibashi et al. [56])

Clinical studies have confirmed the efficacy of pemafibrate in patients with atherogenic dyslipidaemia, either as monotherapy or as add-on to statin treatment (Table 1). In a dose-ranging phase II study in patients with elevated TGs (≥ 200 mg/dL or 2.3 mmol/L) and low HDL-C (< 50 mg/dL in men or < 55 mg/dL in women) [56], treatment with pemafibrate (0.05–0.4 mg daily for 12 weeks), resulted in dose-dependent reduction in TGs (by up to 43% at 0.2–0.4 mg/day) and an increase in HDL-C (by up to 21% at 0.4 mg/day). These lipid-modifying effects were significant compared with placebo but although numerically larger, did not differ significantly from those observed with micronized fenofibrate capsules 100 mg/day (Table 1, Fig. 4). Lipoprotein analysis showed that the increase in HDL-C levels with pemafibrate was attributable to significant increases in the three smaller subpopulations of HDL (medium, small, and very small HDL). Treatment with pemafibrate was also associated with a significant decrease versus placebo in non-HDL-C (by up to 12% at a dose of 0.1 mg twice daily), as well as lipid parameters closely related to TGs, including VLDL-cholesterol (up to 48%), remnant-cholesterol (up to 50%), apoB (up to 9%), apoB48 (up to 56%) and apoCIII (up to 35%). At the highest doses (0.2 and 0.4 mg/day), reduction in VLDL-cholesterol was significantly greater than with fenofibrate 100 mg/day (44 and 48%, versus 26%, respectively) (Table 2) [56]. While there was a slight increase in LDL-C levels (by 5.0–8.9%), apoB and non-HDL-C levels both significantly decreased. Lipoprotein analysis indicated that only large and medium LDL fractions increased during treatment with pemafibrate. There were also dose-dependent increases in apoAI and apoAII (by 9% and 30%, respectively with pemafibrate 0.2 mg twice daily versus 6 and 20% with fenofibrate 100 mg/day) [56].

**Table 1:** Summary efficacy data from published Phase II trials with pemafibrate

Citation no.	Patients	N	Dose (mg/day)	Weeks	Mean ∆ in TG (%)	Mean ∆ in HDL-C (%)
Monotherapy						
[56]	Japanese patients with atherogenic dyslipidaemia^a^	224	Pemafibrate	12		
			0.05		↓30.9 ± 6.9***	↑11.9 ± 2.8***
			0.1		↓36.4 ± 6.6***	↑16.5 ± 2.7***
			0.2		↓42.6 ± 6.7***	↑16.3 ± 2.8***
			0.4		↓42.7 ± 6.7***	↑21.0 ± 2.8***
			Fenofibrate			
			100		↓29.7 ± 6.7	↑14.3 ± 2.8
			Placebo		↑28.5 ± 6.8	↓2.3 ± 2.8
[63]	Japanese patients with atherogenic dyslipidaemia^a^	526	Pemafibrate	12		↑20.3–24.7
			0.1		↓46.3***	
			0.2		↓46.7***	
			0.4		↓51.8***	
			Fenofibrate			↑17.2–26.5
			100		↓38.3***	
			200		↓51.5***	
			Placebo		↓2.7	
Add-on to statin						
[60]	Japanese patients with TG ≥ 2.3 mmol/L	423	Pemafibrate	24		
			0.2		↓46.8 ± 2.6***	↑17.6 ± 17.2***
			0.2/0.4^c^		↓50.8 ± 2.5***	↑16.3 ± 14.6***
			Placebo		↓0.8 ± 3.0	↑4.4 ± 12.7
[60]	Add-on to pitavastatin Japanese patients with TG ≥ 2.3 mmol/L, non-HDL-C ≥ 3.9 mmol/L	188	Pemafibrate	12		
			0.1		↓46.1 ± 3.9***	↑13.6 ± 15.4**
			0.2		↓53.4 ± 3.8***	↑19.7 ± 19.4***
			0.4		↓52.0 ± 3.9***	↑12.7 ± 19.3*
			Placebo		↓6.9 ± 4.0	↑3.4 ± 12.5
[59]	T2DM and dyslipidemia Japanese patients with TG ≥ 1.7 and < 11.3 mmol/L	167	Pemafibrate	24		
			0.2		↓44.3***	
			0.4		↓45.1***	
			Placebo		↓10.8***	
[61]	Caucasian patients, controlled LDL-C and atherogenic dyslipidaemia^b^	408	Pemafibrate	12	↓34.0–54.4***	↑7.4–12.9***
			0.1, 0.2 or 0.4			
[62]	Caucasian type 2 diabetes patients with controlled LDL-C and atherogenic dyslipidaemia^b^	161^d^	Pemafibrate	12	↓44.7–67.4***	NR
			0.1, 0.2 or 0.4			

*HDL-C* high-density lipoprotein cholesterol, *LDL-C* low-density lipoprotein cholesterol, *TG* triglycerides, *T2DM* type 2 diabetes mellitus

** p < 0.01, *** p < 0.001 versus control

^a^TG ≥ 2.3 mmol/L (200 mg/dL) and low HDL-C

^b^TG 1.9–5.7 mmol/L (175–500 mg/dL) and low HDL-C

For both a and b, low HDL-C was defined as < 1.3 mmol/L (50 mg/dL) in men or < 1.4 mmol/L (55 mg/dL) in women

^c^Patients with TG ≥ 1.7 mmol/L (150 mg/dL) at week 8 were uptitrated to 0.4 mg/day from week 12

^d^Subgroup analysis of Kastelein et al. [61]

**Table 2:** Effects of pemafibrate on atherogenic apolipoproteinB-containing lipoproteins in a phase II dose-ranging study Adapted from Ishibashi et al. [56].

Parameter	Placebo, (n = 35)	Pemafibrate (K-877) mg/day				Fenofibrate mg/day 100 (n = 36)
		0.05 (n = 34)	0.1 (n = 37)	0.2 (n = 36)	0.4 (n = 36)	
Total C	0.1 ± 9.8	−2.7 ± 11.4	−6.5 ± 11.9**^†^	−7.0 ± 11.3***^†^	−5.3 ± 12.9*	−6.0 ± 11.8**^†^
Non-HDL-C	0.7 ± 12.8	−5.8 ± 12.4*	−11.8 ± 14.0***^†††^	−12.2 ± 13.8***^†††^	−10.5 ± 14.2***^†††^	−10.1 ± 14.2***^††^
VLDL-C^a^	13.3 ± 38.9	−24.3 ± 24.0***^†††^	−37.3 ± 26.7***^†††^	*−43.8* *±* 24.0***^†††^	*−48.4* *±* 27.5***^†††^	−25.8 ± 29.7***^†††^
LDL-C^a^	−6.3 ± 16.2*	8.9 ± 21.3*^†^	8.3 ± 29.4^†^	5.0 ± 28.0	7.4 ± 26.5^†^	5.3 ± 23.4^†^
Remnant C	38.7 ± 75.7**	−32.3 ± 33.8***^†††^	−42.8 ± 29.4***^†††^	−48.3 ± 28.1***^†††^	−50.1 ± 31.8***^†††^	−31.8 ± 35.0***^†††^
ApoB	−2.0 ± 9.9	−1.4 ± 13.6	−8.9 ± 13.6***^†^	−7.8 ± 15.0**	−8.1 ± 11.6***	−5.7 ± 14.4*
ApoB48	54.6 ± 171.1	−28.4 ± 43.1***^†††^	−43.1 ± 47.1***^†††^	−55.9 ± 25.6***^†††^	−51.2 ± 29.3***^†††^	−37.9 ± 42.9***^†††^
ApoCIII	7.9 ± 27.4	−22.2 ± 14.4***^†††^	−29.0 ± 18.9***^†††^	−34.6 ± 17.7***^†††^	−33.4 ± 19.2***^†††^	−27.2 ± 18.9***^†††^

Data are given as mean ± standard deviation (SD) for the percent change from baseline to week 12

*Apo* apolipoprotein, *C* cholesterol, *HDL* high-density lipoprotein, *LDL* low-density lipoprotein, *VLDL* very low-density lipoprotein

Significantly different from baseline (week 0) * p < 0.05, ** p < 0.01, *** p < 0.001

Significantly different from placebo ^†^ p < 0.05, ^††^ p < 0.01, ^†††^ p < 0.001

Figures in italics: significantly different from fenofibrate p < 0.01

^a^Measured by ultracentrifugation

The TG-lowering effects of pemafibrate were confirmed in combined analysis of Phase II/III studies in 750 patients with elevated TGs (≥ 200 mg/dL or 2.3 mmol/L). Daily doses of pemafibrate (0.1, 0.2 or 0.4 mg) resulted in significantly greater reduction in TGs than fenofibrate 100 mg/day (by 45–52% versus 38%, p < 0.01), although the response was similar to fenofibrate 200 mg daily (decrease by 51%) [57]. Taken together, these findings suggest that pemafibrate has the potential to improve both lipoprotein quality and quantity.

### Non-lipid effects

Interestingly, a clinical pharmacology study in patients with dyslipidaemia showed that pemafibrate 0.4 mg/day increased hepatic glucose uptake, as evaluated by the hyperinsulinaemic-euglycaemic clamp procedure [58]. These findings suggest that pemafibrate may have the potential to ameliorate hepatic insulin sensitivity. Results from Phase II/III trials were also encouraging. In the dose-ranging Phase II study described above [56], treatment with pemafibrate 0.2 mg/day reduced fasting plasma glucose and homeostatic model assessment of insulin resistance (HOMA-IR). Added to this, in a combined analysis of Phase II/III studies including 676 patients with dyslipidaemia, treatment with pemafibrate resulted in significant improvement in fasting plasma glucose, fasting plasma insulin, and HOMA-IR [*Data presented at the 50th European Association for the Study of Diabetes, September 15*–*19, 2014. Vienna, Austria, personal communication from Kowa Company Ltd*.]. Similar findings have been reported in type 2 diabetes patients with dyslipidaemia [59].

Furthermore, there were significant increases in plasma levels of FGF21 after treatment with pemafibrate 0.1–0.4 mg/day for 12 weeks compared with placebo, whereas no effect was observed with fenofibrate 100 mg/day [56, 58]. Taken together, these findings suggest that pemafibrate may have important non-lipid effects, consistent with the pharmacological profile of pemafibrate as a SPPARMα, as shown in vitro and in preclinical studies.

### Add-on to statin

As add-on to statin therapy in 423 Japanese patients with hypertriglyceridaemia (> 200 mg/dL or 2.3 mmol/L), pemafibrate 0.2 mg/day reduced TGs by 47% (versus 0.8% with placebo) (Table 1). Uptitration to 0.4 mg/day in patients with TGs ≥ 150 mg/dL (1.7 mmol/L) at 8 weeks led to a higher proportion of patients attaining the desirable TG level (fasting TGs < 150 mg/dL or 1.7 mmol/L) at week 24 (36.2% versus 29.7%) [60]. A similar response was observed with pemafibrate added to pitavastatin therapy in 188 Japanese patients with hypertriglyceridaemia [60] (Table 1). At the highest dose (0.4 mg/day), there was also significant improvement in fasting blood glucose and HOMA-IR (p < 0.01 and p < 0.05, respectively) [60].

Pemafibrate (0.1–0.4 mg/day) was investigated in a large Phase II study in Europe in patients with atherogenic dyslipidaemia despite well controlled LDL-C levels on stable statin therapy [61]. At the highest dose (0.4 mg/day), TGs were reduced by 54%, remnant-cholesterol (calculated as total cholesterol—LDL-C–HDL-C) by 58%, and apoCIII by 36%, and plasma HDL-C concentration was increased by 13%. Post hoc analysis in patients with type 2 diabetes mellitus at baseline showed improved lipid-modifying efficacy with pemafibrate, with reduction in TGs by up to 67%, remnant cholesterol by 82% and apoCIII by 40% [62].

Taken together, these studies show that pemafibrate (0.2–0.4 mg/day) is effective in the management of hypertriglyceridaemia, with or without low plasma HDL-C concentration, either as monotherapy or against a background of stable statin therapy. There are encouraging data to suggest that pemafibrate improves glucose metabolism, which merits further study.

## Safety and tolerability

Beyond efficacy, safety and tolerability are key considerations for any novel therapy. As already discussed, the SPPARM concept was extended to PPARα in the search for an agent with improved potency and selectivity so as to overcome clinical concerns with the use of fibrates, specifically relating to adverse effects on liver and renal function. Evidence from the phase II/III study [63] showed that pemafibrate was well tolerated, with adverse event rates similar to or lower than those reported for placebo or fenofibrate. Notably, pemafibrate was associated with a lower rate of abnormal liver function tests compared with fenofibrate; only one patient discontinued due to this event compared with 3 and 11 patients allocated fenofibrate 100 mg and 200 mg, respectively.

Importantly, the available safety data show that treatment with pemafibrate does not appear to affect renal function parameters. In the integrated analysis of phase II/III Japanese studies, there was no change in estimated glomerular filtration rate (eGFR), whereas a significant decline in eGFR was observed with fenofibrate treatment over 12 weeks (p < 0.001) [57], consistent with previous findings from both the Fenofibrate Intervention and Event Lowering in Diabetes (FIELD) and ACCORD studies [64, 65]. In a phase II study in Europe [61, 62], minor effects on changes in serum creatinine observed over 12 weeks with pemafibrate were considered clinically negligible or not associated with treatment, even in patients with type 2 diabetes mellitus at baseline. A small increase in serum homocysteine levels (by 2.4 μmol/L) was noted at a dose of 0.4 mg/day compared with placebo, although the clinical relevance of this is indeterminate [61].

In conclusion, the available safety experience with pemafibrate is encouraging, with no clinically meaningful deleterious effects on renal or hepatic function. However, long-term data in real world practice are needed to fully evaluate the safety profile of this novel agent.

## Unanswered questions

The available data show the benefits of applying the SPPARM concept to PPARα, showing effective lowering of TGs, as well as other atherogenic measures, notably VLDL- and remnant cholesterol, in statin-treated patients with atherogenic dyslipidaemia. The safety profile of pemafibrate appears promising, with no evidence of clinically meaningful adverse effects on renal or hepatic function during treatment for up to 24 weeks, thus reinforcing the value of improved selectivity with this SPPARMα.

The key question, however, is whether this novel SPPARMα impacts the high residual risk of cardiovascular events that persists in statin-treated patients with atherogenic dyslipidaemia, especially those with concomitant type 2 diabetes mellitus. Both the FIELD and ACCORD Lipid trials have failed to provide definitive answers with the use of fenofibrate, largely due to methodological reasons. The FIELD trial was initiated before statin use was considered first-line for prevention of CVD; indeed, the study criteria excluded patients with an indication for lipid-lowering therapy. The study population was at low to moderate global CVD risk, 75% had no prior CVD and only about one-third of patients had the characteristic atherogenic dyslipidaemic profile associated with type 2 diabetes (i.e., low HDL-C and elevated triglycerides) [66]. While in ACCORD Lipid all patients received concomitant simvastatin treatment, and the study population was at higher cardiovascular risk than in FIELD (37% had previous CVD), median triglycerides (164 mg/dL or 1.8 mmol/L) were below that indicated for definition of atherogenic dyslipidaemia [67].

To address these remaining uncertainties regarding PPARα-targeted agents, PROMINENT (Pemafibrate to Reduce cardiovascular OutcoMes by reducing triglycerides IN diabetic patiENTs) has been initiated. This study aims to recruit 10,000 patients with type 2 diabetes mellitus and elevated TGs (≥ 200 mg/dL or 2.3 mmol/L and < 500 mg/dL (5.7 mmol/L) and low HDL-C (≤ 40 mg/dL or 1.0 mmol/L), with and without established CVD [68]. Patients will be randomised to treatment with pemafibrate 0.4 mg/day or placebo, against a background of aggressive, standard of care management of cardiovascular risk factors including treatment with high-intensity statins. The primary study endpoint is a composite of nonfatal myocardial infarction, nonfatal ischaemic stroke, hospitalisation for unstable angina requiring unplanned coronary revascularisation, or cardiovascular death [68]. Anticipating an average follow-up of 4 years, we can expect answers to this question by early 2022.

Another area of potential therapeutic interest is in the management of non-alcoholic fatty liver disease (NAFLD). Already there are promising results from a phase II b trial with elafibranor (GFT-505), a PPAR-α/PPAR-δ agonist, that improving atherogenic dyslipidaemia and insulin resistance may have benefit in patients with non-alcoholic steatohepatitis (NASH) [69]. With a profile including reduction in TGs and remnant cholesterol, upregulation of FGF21, and the lack of clinically meaningful effects on hepatic function, pemafibrate may have a possible role in NAFLD, specifically NASH. Indeed, in animal studies, pemafibrate appeared to ameliorate diet-induced NASH [70], by modulation of lipid turnover and energy metabolism, as well as NAFLD induced by a methionine-choline-deficient diet, by increased expression of fatty acid β-oxidation genes [71].

Finally, potential benefits of pemafibrate on microvascular complications of type 2 diabetes, notably diabetic retinopathy, previously reported for fenofibrate [72, 73], merit investigation. While the pathogenesis of diabetic retinopathy is still incompletely understood, evidence suggests a role for diabetes-induced down-regulation of PPARα [74], as well as the involvement of inflammatory pathways, especially in obese individuals [75]. A nested study of PROMINENT will investigate this issue. Studies in *db/db* mice also showed that pemafibrate inhibited the diacylglycerol-protein kinase C-NAD(P)H oxidase pathway, resulting in suppression of lipid accumulation and oxidative stress in the kidney [76]. Thus, given the improved selectivity and potency of this SPPARMα agent, as well as evidence of anti-inflammatory effects, pemafibrate may offer potential for ameliorating this important, disabling diabetic microvascular complication.

## Conclusions

Atherogenic dyslipidaemia, prevalent among patients with type 2 diabetes and obesity, is a contributor to lipid-related residual cardiovascular risk. Accumulating evidence has led to renewed thinking about the importance of targeting this dyslipidaemia, in particular elevated TGs, to reduce this risk. Among current therapeutic options, fibrates are the logical option, but have relatively low potency and selectivity for PPARα. Extension of the SPPARM concept to PPARα has led to the development of pemafibrate, which in preclinical studies, has shown improved potency and selectivity compared with currently available fibrates. In clinical trials, pemafibrate was effective in the management of atherogenic dyslipidaemia, either as monotherapy or add-on to statin treatment. There were no clinically meaningful adverse renal effects, including no elevation in serum creatinine, and no significant effects on hepatic function. Long-term data are needed, however, for definitive evaluation of the benefit versus risk of this novel SPPARMα for reduction of residual cardiovascular risk. Finally, investigation of a possible role for pemafibrate in the management of microvascular complications of diabetes, notably diabetic retinopathy, as well as in NAFLD, may be merited. A novel approach to addressing residual cardiovascular risk may be well within our grasp.

## Abbreviations

ABCA1: ATP-binding cassette transporter ABCA1 (member 1 of human transporter sub-family ABCA)
ABCG1: ATP-binding cassette sub-family G member 1
ACCORD: Action to Control Cardiovascular Risk in Diabetes
Apo: apolipoprotein
CREBH: cyclic AMP responsive element-binding protein
CVD: cardiovascular disease
eGFR: estimated glomerular filtration rate
ENPEP: glutamyl aminopeptidase
FGF21: fibroblast growth factor 21
FIELD: Fenofibrate Intervention and Event Lowering in Diabetes
FOURIER: Further Cardiovascular Outcomes Research With PCSK9 Inhibition in Subjects With Elevated Risk
HDL-C: high-density lipoprotein cholesterol
HMGCS2: 3-hydroxy-3-methylglutaryl-CoA synthetase 2
HOMA-IR: homeostatic model assessment of insulin resistance
IMPROVE-IT: Examining Outcomes in Subjects With Acute Coronary Syndrome: Vytorin (Ezetimibe/Simvastatin) vs Simvastatin
LDL-C: low-density lipoprotein cholesterol
LPL: lipoprotein lipase
MBL2: mannose-binding lectin 2
MCP-1: monocyte chemoattractant protein-1
NAFLD: non-alcoholic fatty liver disease
NASH: non-alcoholic steatohepatitis
NCoR1 and NCoR2: nuclear receptor co-repressors 1 and 2
PPARα: peroxisome proliferator-activated receptor alpha
PPRE: peroxisome proliferator-activated receptor response element
PROMINENT: Pemafibrate to Reduce cardiovascular OutcoMes by reducing triglycerides IN diabetic patiENTs
REVEAL: Randomized EValuation of the Effects of Anacetrapib Through Lipid-modification
RXR: retinoid X receptor
SPPARMα: selective PPARα modulator
SR-B1: scavenger receptor B1
TG: triglyceride
VCAM-1: vascular cell adhesion molecule 1
VLDL: very low-density lipoproteins
